# Biological compatibility of oxidized cellulose vs. porcine gelatin to control bleeding in liver lesions in rats

**DOI:** 10.1590/ACB361101

**Published:** 2022-01-05

**Authors:** Maria de Lourdes Pessole Biondo-Simões, Jaqueline Alves Zwierzikowski, Juliane Castro Duarte Antoria, Sérgio Ossamu Ioshii, Rogério Ribeiro Robes

**Affiliations:** 1 Full Professor. Department of Surgery. Postgraduate Program in Clinical Surgery - Universidade Federal do Paraná – Curitiba (PR), Brazil.; 2 Graduate student. School of Medicine - Universidade Federal do Paraná – Curitiba (PR), Brazil.; 3 Full Professor. Department of Pathology - Universidade Federal do Paraná (UFPR) and Pontifícia Universidade Católica do Paraná - Curitiba (PR), Brazil.; 4 Physician. Veterinary Hospital. Fellow Master degree. Postgraduate Program in Clinical Surgery - Universidade Federal do Paraná – Curitiba (PR), Brazil.

**Keywords:** Liver, Trauma, Regeneration, Wound Healing, Hemostatics, Models, Animal

## Abstract

**Purpose:**

To compare biological compatibility, hemostasis, and adhesion formation between oxidized regenerated cellulose and lyophilized hydrolyzed porcine collagen in liver trauma.

**Methods:**

Forty male Wistar rats constituted two groups: group A (oxidized cellulose) and group B (lyophilized hydrolyzed collagen). Standardized liver trauma was made, and the hemostatic agent was applied. Animals in subgroups A7 and B7 were submitted to euthanasia and relaparotomy after seven days, and in subgroups A14 and B14 after 14 days. Macroscopic and microscopic results were evaluated.

**Results:**

There was no fluid in the cavity in any of the animals, and adhesions were present in all of them. In the analysis after seven days, the adhesions were grades 3 or 4 and consisted of omentum, small intestine, and abdominal wall (p<0.05). In both groups, the mesh was surrounded by a capsule, which was not observed after 14 days. In the evaluation after 14 days, adhesions were grades 2 or 3 (p>0.05). The microscopic examination showed subacute and chronic reactions, in both groups and in both timepoints, with similar frequency. The intensity of fibrosis always presented positive scores. Microabscesses and xanthomatous macrophages were observed in both groups.

**Conclusions:**

There was no superiority of one agent over the other.

## Introduction

Every year, 5.8 million people die from trauma worldwide. It is the most frequent cause of death in individuals under 40 years of age^1^.

The abdomen is commonly afflicted with penetrating and blunt trauma. The liver, mainly due to its size and anatomical location, is frequently affected^2^^-^ 4. Liver trauma accounts for 5% of all admissions to emergency rooms^5^.

Most liver traumas, graded as minor or moderate (grades I or II), usually require little or no intervention and can be treated by nonoperative management (NOM). More severe traumas (grades III or IV) can be treated with NOM, but they are predominantly managed surgically, while grades V and VI traumas almost always require operative treatment.

The main objectives of intraoperative management are control of bleeding and of biliary leakage, debridement of devitalized tissue, and proper drainage^2^^,^ 4 ^,^ 6.

Some surgical maneuvers for the management of liver injury are:

- Pringle maneuver^6^;
- Perihepatic packing^7^;
- Mesh wrapping^7^;
- Balloon tamponade^7^;
- Hepatorrhaphy, using deep parenchymal sutures^7^;
- Hepatotomy (finger fracture, allowing direct ligation or clipping of the vessels)^2^^,^ 4 ^,^ 6 ^,^ 8;
- Hepatic debridement with removal of devitalized tissue to minimize postoperative sepsis and secondary bleeding^9^;
- Lobectomy^8^^,^ 10;
- Selective hepatic artery ligation^7^;
- Use of local hemostatic agents^7^.

Uncontrolled hepatic bleeding is directly related to mortality, which can reach 54% of the cases, since it is richly vascularized and its sinusoids lack smooth muscle, that is important for vasoconstriction and hemostasis^6^.

Hemostatic agents are tools that can help control bleeding^11^. Several agents are available in the market in different forms and exerting their effects in various ways. They can improve primary hemostasis, stimulate fibrin formation, or inhibit fibrinolysis^11^. Some are composed of a procoagulant substance combined with a collagen matrix. Others use this matrix to provide a model for the endogenous coagulation cascade, achieving hemostasis^12^.

Oxidized regenerated cellulose is a local, biodegradable hemostatic derived from the controlled oxidation of cotton cellulose^11^^,^ 13. It is the most used hemostatic material in clinical practice, and its absorption takes about two to three weeks^3^^,^ 14. Its mechanism of action, although not yet fully understood, is mainly based on a mechanical compressive effect. In addition, polyanhydroglucuronic acid, present in oxidized cellulose, with a pH around 3, facilitates hemostasis by denaturing blood proteins and prevents bacterial growth^10^^,^ 11 ^,^ 13 ^,^ 14.

Porcine gelatin is made from purified pigskin gelatin and it is available as an absorbable gelatin sponge and as a compression sponge^13^. It is insoluble in water and expands when in contact with liquids, absorbing them and storing up to 45 times its weight, promoting passive mechanical hemostasis. It can be used alone or in combination with a saline solution or topical thrombin, remaining in the body for four to six weeks up to its absorption^14^.

The purpose of this study was to compare biological compatibility, achievement of hemostasis and adhesion formation of two hemostatic agents: oxidized regenerated cellulose, of vegetal origin, and lyophilized hydrolyzed porcine collagen, of animal origin.

## Methods

The experiments were performed in accordance with the Brazilian Guidelines for the Care and Use of Animals for Scientific and Didactic Purposes, edited by the Ministry of Science, Technology and Innovation, National Council for the Control of Animal Experimentation (CONCEA), in 2013, and the Federal Law No. 11.794, of October 8, 2008. The project was approved by the Ethics Committee on Animal Use of the Sector of Biological Sciences of the Universidade Federal do Paraná (CEUA/BIO – UFPR) on September 17, 2019, receiving the No. 1,317.

Forty male Wistar rats – *Rattus norvegicus albinus*, Rodentia, Mammalia –, 140 days old and weighing 450.8 ± 36.6 g were randomly allocated into two main groups: group A and group B. Group A was treated with oxidized regenerated cellulose (Surgicel®), and group B with lyophilized hydrolyzed porcine collagen (Hemospon®). The first 10 animals from each group were allocated to subgroups A7 and B7, and the other 10 to groups A14 and B14. Subgroups A7 and B7 were evaluated on the seventh postop day (POD) and subgroups A14 and B14 on the fourteenth.

The animals were maintained in the Surgical Technique and Experimental Surgery Laboratory with relative humidity proper to the environment and controlled temperature (20 ± 2°C), in a light-dark cycle of 12 h, and received water and feed (standard for the species) *ad libitum*.

After quarantine, on the day scheduled for the intervention, the animals were fasted for 2 h preoperatively.

Anesthesia and analgesia were conducted by a veterinarian. Anesthesia was initiated with a pre-anesthetic intramuscular injection of ketamine hydrochloride 50 mg/kgcombined with xylazine hydrochloride 2 mg/kg. Anesthetic induction was performed by inhalation with 1% isoflurane, and maintenance with the same drug at 1.5% under mask, associated with 100% oxygen.

The anesthetized rats had their ventral region shaved and were identified. This was followed by antisepsis with 1% chlorhexidine digluconate. Then, the rats were positioned on a surgical board and submitted to a 4-cm laparotomy, followed by a lesion in the left lateral lobe of the liver, starting at the border and compromising the entire thickness of the parenchyma, made with a 15-blade scalpel.

**Figure 1:**
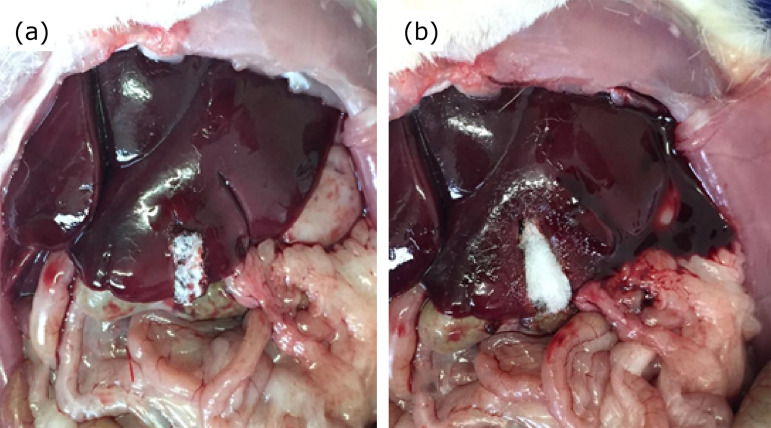
Aspect observed after application of the hemostats. **(a)** Oxidized regenerated cellulose; **(b)** lyophilized hydrolyzed porcine collagen.

Hemostasis was promoted with application of hemostatic sponge of oxidized regenerated cellulose (Surgicel®) in group A or lyophilized hydrolyzed porcine collagen (Hemospon®) in group B (Fig. 1).

After attesting hemostasis, the abdominal wall was sutured in two planes: peritoneum-muscle-aponeurotic plane, with continuous running 4-polyglactin 910 suture; and then skin, with continuous running 4-nylon monofilament suture.

After recovery from anesthesia, the animals were put back in their original boxes with food and water available. Postoperative analgesia was performed by intramuscular injection of dipyrone sodium monohydrate 50 mg/kg, maintained every 12 h.

To verify the results, the animals were anesthetized and euthanized by a veterinarian, according to the protocol described in the CONCEA Euthanasia Practice Guidelines, Resolution no. 37 of the Ministry of Science, Technology, Innovation and Communication, of February 15, 2018.

Once death was confirmed, the abdominal cavity was opened with an inverted C-shaped incision, so that the ventral abdominal wall could be folded to the right, without damaging any adhesions that might exist.

The presence of fluid in the cavity and the presence and intensity of adhesions were evaluated. Structural criteria such as width (thin or thick), stability (firm or loose), constitution, and dimension were used for classification, as well as the Granat *et al*. scale^15^.

Then, the hepatic segment of interest was resected with the adhesions, if present, fixated in 10% formalin and sent to pathology.

The embedded material underwent 4-μm thick section,s that were assembled on slides and stained with hematoxylin-eosin (HE). The analysis was performed by reading five fields on each slide. Edema, congestion, granulation tissue, fibrosis, polymorphonuclear, monomorphonuclear, and foreign body giant cells were analyzed. This information provided the knowledge of the type of inflammatory reaction and its quantification, allowing us to obtain the final score of each group, according to Vizzotto Junior *et al*.’s method^16^. The presence of microabscesses and xanthomatous macrophages was evaluated and quantified as absent or present in small, moderate, or large quantities.

The results were tabulated and submitted to statistical analysis. Median, mean, standard deviation of the mean, maximum and minimum values were used to evaluate measurement-related variables. As the results did not provide a Gaussian curve, the non-parametric Mann-Whitney test was used for the analysis. Fisher’s exact test was used for the frequency measurements. The significance level considered for the null hypothesis to be rejected was p ≤ 0.05.

## Results

No animals were lost during the experiment. Although the time required to achieve hemostasis was not being evaluated, it was noticeable that oxidized regenerated cellulose promoted hemostasis faster than lyophilized hydrolyzed porcine collagen.

**Figure 2:**
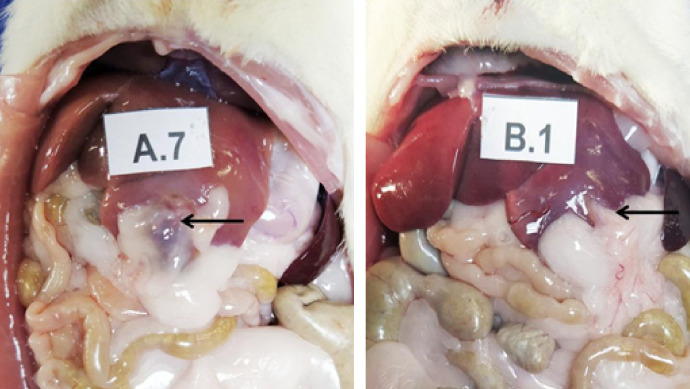
Examples of adhesions found after seven days of evolution. **(A.7)** Oxidized regenerated cellulose group; **(B.1)** lyophilized hydrolyzed porcine collagen group greater *omentum* adhered to the site of application of the hemostats.

**Figure 3:**
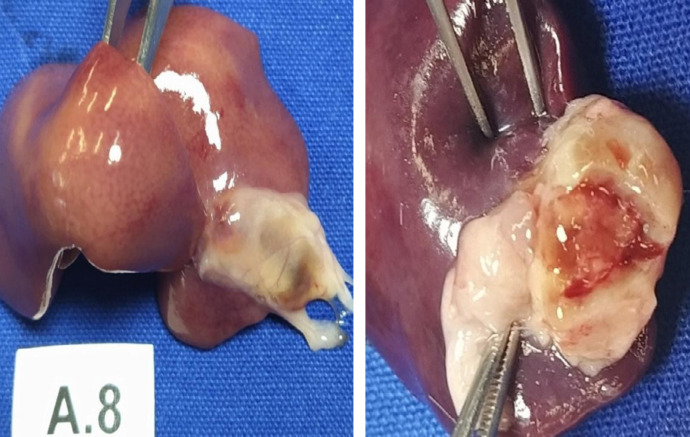
Encapsulated hemostatic material.

Adhesions were present in all animals of both groups (Fig. 2). The liver lesion site showed thicker, wider adhesions that could involve adjacent organs at the POD 7 evaluation. In all cases, the adhesions involved the greater omentum, but in some it was possible to find loops of small intestine and even abdominal wall. The mesh used was involved by a capsule in seven of the 10 animals of group A7 and in five of the 10 animals of group B7 (Fig. 3). In POD 14, this condition was not observed. All adhesions observed at the seventh day, both in groups A and B, were grades 3 or 4, with no significant difference between the groups (p = 0.6563). In the evaluation after 14 days, the intensity of the adhesions was lower, with grades 2 and 3 in both groups, without differences between them (p = 0.3698).

**Figure 4:**
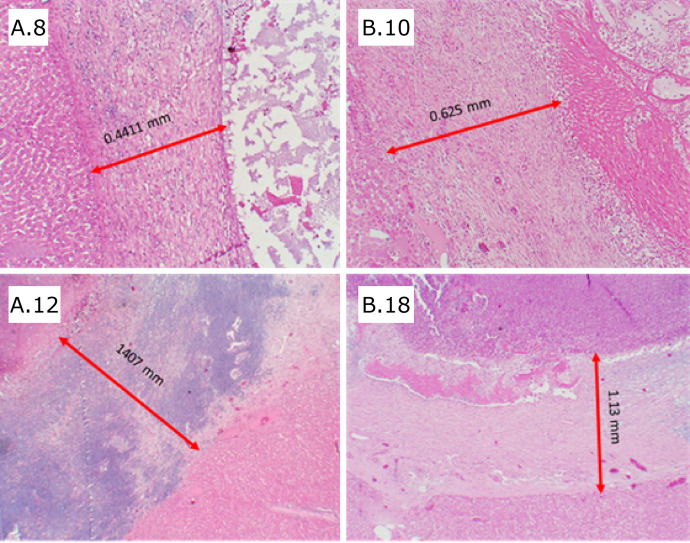
Photomicrographs showing the encapsulated hemostatic material and the thickness of the capsules, in millimeters. **(A.8)** Histological section of the liver of animal no. 8, group A, after seven days; **(B.10)** histological section of the liver of animal no. 10, group B, after seven days; **(A.12)** histological section of the liver of animal no. 12, group A, after 14 days; **(B.18)** histological section of the liver of animal no. 18, group B, after 14 days.

The result of the inflammatory score showed subacute and chronic reactions in both groups and in both timepoints, with similar frequencies (seven days, p=1; and 14 days, p=0.580).

n photomicrographs, the hemostatic material was visualized with amorphous characteristics, surrounded by a fibrotic capsule (Fig. 4). The intensity of fibrosis always showed positive scores, similarly between the groups, both after seven (p=0.303) and 14 days (p=0.170). The mean capsule thickness, measured in four fields for each slide, showed no significant difference.I

Microabscesses were observed at both timepoints with no significant difference between groups A and B after seven (p = 0.211) or 14 days (p = 0.170).

Xanthomatous macrophages were detected in both groups and at both timepoints. At the POD 7, they were present in small or moderate quantities (p=0.170). After 14 days, they were seen in moderate quantity in group B and in large quantity in group A (p=0.070).

## Discussion

In a report by Sikhondze *et al*.^17^, liver injuries occurred in 22% of the cases of abdominal trauma, and severe bleeding from these injuries was one of the main causes of mortality in these patients.

To help manage this situation, one of the available alternatives is the use of hemostatic agents as adjuvants. The most used ones are oxidized regenerated cellulose, microfibrillar collagen, lyophilized hydrolyzed porcine collagen, fibrin sealant and fibrin-gelatin^7^^,^ 14 ^,^ 18.

This study sought to evaluate the biocompatibility of two hemostatic materials that are widely available for use in surgical procedures: oxidized regenerated cellulose (Surgicel®) and a lyophilized hydrolyzed porcine collagen (gelatin) hemostatic sponge (Hemospon®).

Although the analysis of time to or efficacy of hemostasis was not the object of this study, it was possible to see that oxidized regenerated cellulose promoted hemostasis faster. This parameter has controversial evidences in literature. Fontes *et al*.^19^ stated that there is no superiority of hemostasis between equine collagen sponge and oxidized regenerated cellulose. However, Xu *et al*.^20^, in a study with 92 patients undergoing spondylodesis, concluded that the cellulose-based product had greater hemostatic effect than the control group (gelatin-based products), and MacDonald *et al*.^21^, studying liver injuries in pigs and comparing various hemostatic products, reported superiority in effectiveness and time to homeostasis with oxidized cellulose. Therefore, further comparative studies are necessary to evaluate the control of hemostasis and the required time to achieve it. It is noteworthy that in this study both analyzed agents promoted good hemostasis, as demonstrated by the absence of free fluid or organized material in the abdominal cavity.

It is important to clarify that mechanical hemostatic agents were addressed in this study, because they are the cheapest and most widely available ones. However, biological hemostatic agents, which use thrombin (bovine or human), fibrin sealants, polysaccharide-based products, and hemostatic agents with albumin or based on inorganic substances have shown superiority in achieving hemostasis compared to mechanical hemostatic agents^12^^,^ 13 ^,^ 22.

In this study, adhesions were formed with the use of both hemostatic agents, mainly with the greater omentum and, less frequently, with the small intestine. It is important to consider that their formation over the material used may have helped to control the bleeding.

The pathogenesis of adhesions occurs by aberrant recovery of the healing processes of the peritoneum; surgical trauma and inflammatory or infectious processes can lead to their formation. With the evolution of the healing process, the fibrinolysis mechanisms (plasmin degrading fibrin and activating extracellular matrix metalloproteinases) cause complete depletion of local fibrin deposits generated post-intervention, resulting in complete local recovery^23^. Mavigök *et al*.^24^ evaluated adhesion formation with the use of different homeostatic methods in abdominopelvic surgical procedures in an animal model and found no significant difference when using gelatin sponge compared to oxidized regenerated cellulose.

The inflammatory process triggered by the absorption of topical hemostatic agents depends on the presence of macrophages and generates inflammatory granulomatous reaction^22^^,^ 23. The degree of inflammatory reaction indicates the level of tissue response to the foreign agent. In this study, inflammatory reactions classified as subacute and chronic were found in both groups and at both timepoints evaluated, with no significant difference between them. The formation of a capsule was perceived both macroscopically and microscopically. The capsules were similar in thickness and had xanthomatous macrophages and microabscesses present with the same frequency, which allows us to assume that the intensity of the inflammatory response triggered by the hemostatic agents evaluated was similar.

Regarding inflammatory response, literature has controversial findings. While Fontes *et al*.^19^ found no significant difference in the intensity of the inflammatory reaction when using oxidized regenerated cellulose and gelatin, Gabrielli *et al*.^25^ reported that the inflammatory process was more intense and persistent when using oxidized regenerated cellulose than when using porcine gelatin. In a comparative study with different hemostatic agents, Genyk *et al*.^22^ found worse biocompatibility with the cellulose material, resulting in worse resorption and worse outcomes of inflammation and infection, thus questioning its use in liver resection surgery.

In literature, there are reports of foreign body reactions, granulomas, and even abscesses when oxidized cellulose is used^12^^,^ 22 ^,^ 26. Kim *et al*.^27^ found no abscesses or infectious changes, nor signs of severe inflammation at microscopy when using oxidized regenerated cellulose in abdominal surgery in pigs. Yoon *et al* found similar results when investigating the use of oxidized regenerated cellulose in an animal model^28^.

While oxidized cellulose has acidic pH, which makes it more resistant to infection, porcine gelatin has neutral pH. Yoon *et al*.^28^ described increased incidence of infection, granuloma, and fibrosis formation with the use of gelatin.

In this study, we did not detect significant differences in biological compatibility, hemostasis, or adhesion formation between the two evaluated hemostatic agents. Thus, it is possible to say that there was no superiority of one hemostat over the other. Costs and availability are also important factors, as porcine gelatin is sold at approximately one third of the price of oxidized regenerated cellulose. This disparity should be taken into consideration when using these agents in health services.

## Conclusion

There was no significant difference in terms of biological compatibility, hemostasis or adhesion formation between porcine gelatin and oxidized regenerated cellulose. Therefore, no superiority of one hemostatic agent over the other was found.
